# Supplementary material for: The IGF-Independent Role of IRS-2 in the Secretion of MMP-9 Enhances the Growth of Prostate Carcinoma Cell Line PC3
Source: Int J Mol Sci. 2023 Oct 11;24(20):15065. doi: 10.3390/ijms242015065 (PMC10606031; doi:10.3390/ijms242015065)
Supplement: Supplementary file 1 [file ijms-24-15065-s001.zip › ijms-2626295-supplementary.pdf]

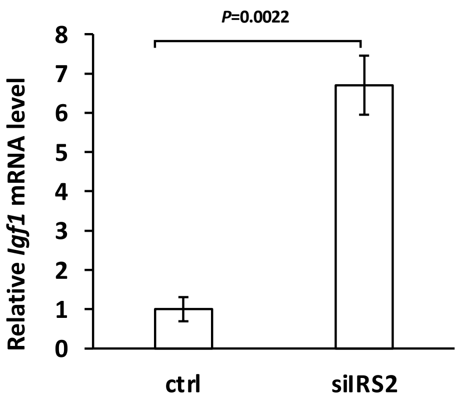

Supplementary Figure S1. Analysis of IGF-I mRNA level
PC3 cells are transfected with siRNA against scramble (ctrl) or IRS-2. The *Igf1* mRNA level was then examined by real-time PCR analysis. Bar graphs are presented as fold-change of columns on the left (ctrl). Bar: mean ± S.E.M., p = 0.0022, n=3, Student's *t*-test.

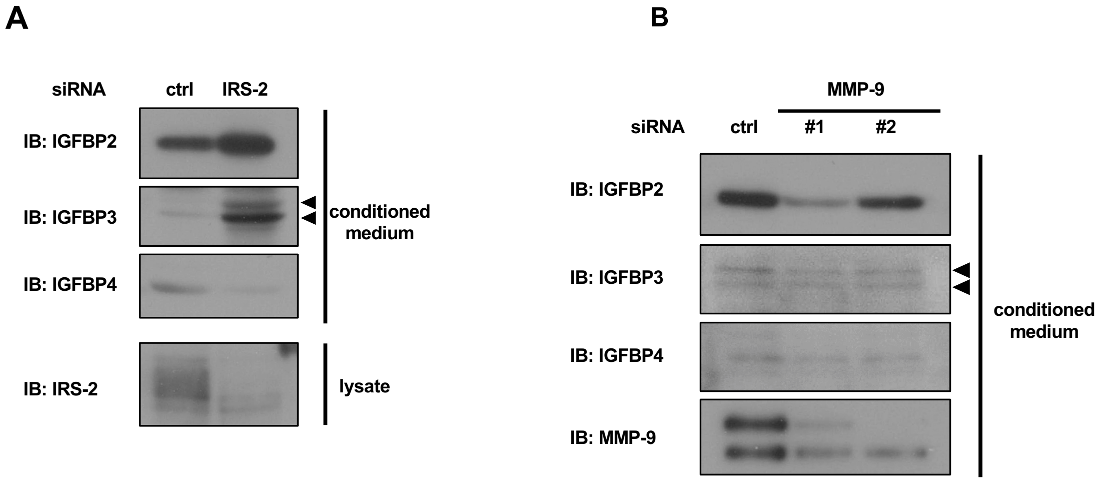

Supplementary Figure S2. Analysis of IGFBPs protein level in the conditioned medium

(A): PC3 cells are transfected with siRNA against scramble (ctrl) or IRS-2. Each cell was cultured in the serum-free medium and conditioned medium was collected. Conditioned medium was subjected to the immunoblotting analysis with indicated antibodies. **(B):** PC3 cells are transfected with siRNA against scramble (ctrl) or MMP-9. Each cell was cultured in the serum-free medium for 24 hours and conditioned medium was collected. Conditioned medium was subjected to the immunoblotting analysis using indicated antibodies.

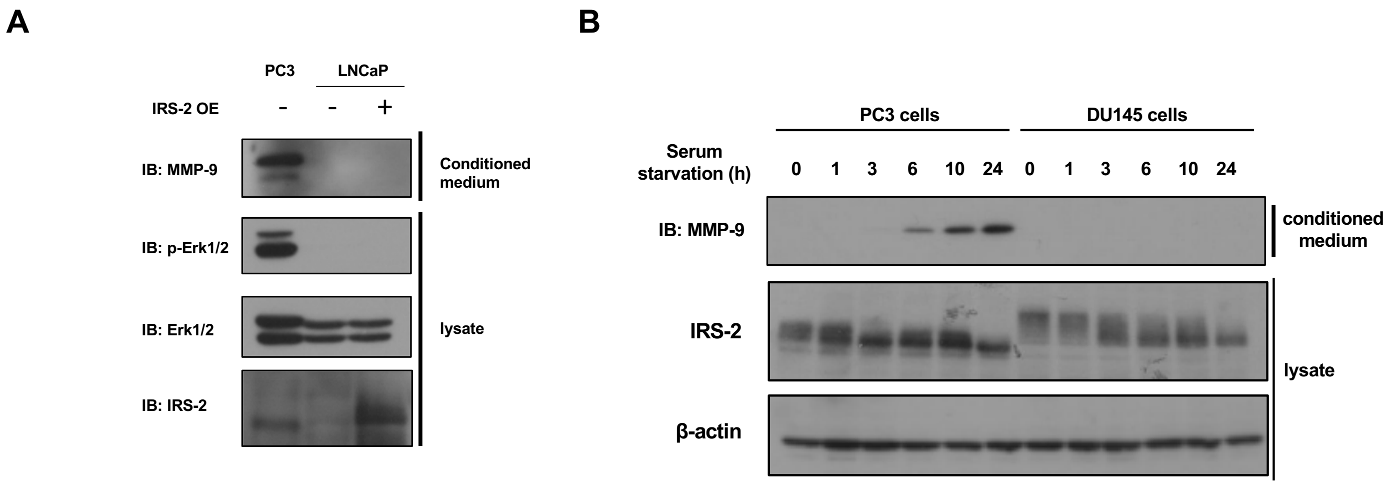

Supplementary Figure S3. MMP-9 secretion levels in some prostate cancer cells.

LNCaP cells are transfected with mock (-) or IRS-2 expressing plasmids (+). PC3 or LNCaP cells were serum-starved for 24 h. Cell lysates or conditioned medium was subjected to immunoblotting using the indicated antibodies. DU145 prostate cancer cells were serum-starved for 24 h. Cell lysates or conditioned medium was subjected to immunoblotting analysis using the indicated antibodies.
